# Relationship between Hyperuricemia and Haar-Like Features on Tongue Images

**DOI:** 10.1155/2015/363216

**Published:** 2015-04-15

**Authors:** Yan Cui, Shizhong Liao, Hongwu Wang, Hongyu Liu, Wenhua Wang, Liqun Yin

**Affiliations:** ^1^School of Computer Science and Technology, Tianjin University, 92 Weijin Road, Nankai District, Tianjin 300072, China; ^2^Department of Common Required Courses, Tianjin University of Traditional Chinese Medicine, 312 Anshanxi Road, Nankai District, Tianjin 300193, China; ^3^College of Traditional Chinese Medicine, Tianjin University of Traditional Chinese Medicine, 312 Anshanxi Road, Nankai District, Tianjin 300193, China; ^4^Wuqing Chinese Medicine Hospital, 10 Jichang Road, Wuqing District, Tianjin 301700, China

## Abstract

*Objective*. To investigate differences in tongue images of subjects with and without hyperuricemia. *Materials and Methods*. This population-based case-control study was performed in 2012-2013. We collected data from 46 case subjects with hyperuricemia and 46 control subjects, including results of biochemical examinations and tongue images. Symmetrical Haar-like features based on integral images were extracted from tongue images. *T*-tests were performed to determine the ability of extracted features to distinguish between the case and control groups. We first selected features using the common criterion *P* < 0.05, then conducted further examination of feature characteristics and feature selection using means and standard deviations of distributions in the case and control groups. *Results*. A total of 115,683 features were selected using the criterion *P* < 0.05. The maximum area under the receiver operating characteristic curve (AUC) of these features was 0.877. The sensitivity of the feature with the maximum AUC value was 0.800 and specificity was 0.826 when the Youden index was maximized. Features that performed well were concentrated in the tongue root region. *Conclusions*. Symmetrical Haar-like features enabled discrimination of subjects with and without hyperuricemia in our sample. The locations of these discriminative features were in agreement with the interpretation of tongue appearance in traditional Chinese and Western medicine.

## 1. Introduction

Hyperuricemia is a metabolic disorder in which the body produces excessive uric acid and fails to excrete it. Excess dietary purines (e.g., from meat and certain seafood) play a significant role in hyperuricemia and contribute to gout [1]. More precisely, hypoxanthine is considered to be an important factor contributing to hyperuricemia [2]. Decreased uric acid excretion is most commonly attributed to genetic factors and medications [3, 4]. Although the mechanism remains unknown, many studies have found relationships between hyperuricemia or urinary abnormalities and impaired kidney function [5, 6]. Thus, impaired kidney function is considered to be a risk factor for hyperuricemia [7]. In turn, hyperuricemia is considered to be a risk factor for severe diseases that can impact quality of life and lead to disability and even death, including coronary heart disease, hypertension, stroke, and insulin resistance [8–11].

With rapid economic development, daily diet and healthcare in China have improved. The prevalence of hyperuricemia has increased with dietary purine content; according to a meta-analysis conducted in 2011, it was 21.6% among males and 8.6% among females in China [12]. For comparison, the prevalence of hyperuricemia in the United States was only 12.7% in 2010 [13]. The high prevalence of hyperuricemia renders its accurate diagnosis critical.

Serum uric acid (SUA) concentration analysis is the gold standard for hyperuricemia diagnosis. However, this method necessitates invasive blood sample collection and biochemical examination, which are time consuming and laborious and risk patient injury. The development of a rapid, simple, noninvasive method would thus improve the diagnostic procedure for hyperuricemia.

Tongue images have been applied as inexpensive and noninvasive means of diagnosing several diseases, such as stroke and appendicitis [14–16]. Wang et al. [17] statistically analyzed features extracted from tongue images, defining 12 image classes. Other statistical methods, such as Bayesian networks and a bagging tree algorithm have been applied to tongue image analysis [15, 16]. However, these studies did not employ case-control designs that would have avoided bias introduced by age and sex differences in tongue features. Jung et al. [18] performed a case-control study to examine differences in color distribution on tongue images between subjects with and without sleep disorders, but the diagnostic criteria used in this study were based on the physician's judgment, rather than biochemical examination. Western medical studies have found that tongue appearance (coloration and coating) is related to kidney diseases or conditions, such as renal adenocarcinoma tongue metastasis and kidney transplantation [19, 20]. Traditional Chinese medicine (TCM) studies have also found that tongue image characteristics can reflect renal deficiency [21, 22].

The present case-control study was performed to identify tongue image features useful for the diagnosis of hyperuricemia. A series of symmetrical Haar-like features, which have been applied successfully to face detection [23], were extracted from tongue images from subjects with and without hyperuricemia (diagnoses were confirmed biochemically). We sought to identify independently useful and readily interpretable Haar-like features for the diagnosis of hyperuricemia.

## 2. Subjects and Methods

### 2.1. Subjects and Examination

Between August 2011 and June 2012, outpatients from Wuqing Chinese Medicine Hospital, a medical examination center of teaching hospital affiliated with the Tianjin University of Traditional Chinese Medicine (TJUTCM), participated in this study. All participants provided informed consent and this study was approved by the medical ethics committee of TJUTCM. Adults from all age groups were included to avoid bias introduced by uneven age distribution. Based on data from medical records accessed through the hospital's health information system, subjects with diseases impacting the appearance of the tongue, such as hypertension, diabetes, and cancer, were excluded. Those with dyed and scraped tongue fur, as determined by outpatient interviews, were also excluded.

The diagnostic criteria for hyperuricemia in males and females were SUA >416 *μ*mol/L (7 mg/dL) and SUA >357 *μ*mol/L (6 mg/dL), respectively [24]. An image of each subject's tongue was acquired using a YM-III tongue image analyzer (http://tjtzym.com/tongue.html).

Case and control subjects were matched 1 : 1 by age (within 1 year) and sex to exclude the impacts of these covariates and improve the value of the empirical data [25, 26]. Two-tailed* t*-tests for samples with equal and unequal variance were used to confirm similarity in age and difference in SUA value, respectively, between case and control subjects.

### 2.2. Image Processing and Feature Selection

**Figure 1:**
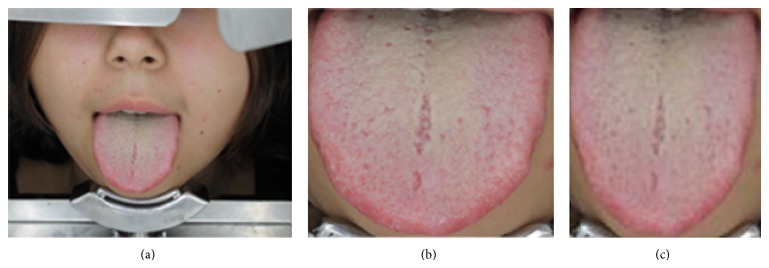
Image processing procedure. (a) Original image acquired by the tongue analyzer. (b) Rectangular area containing the tongue segmented from the original image. (c) Final image scaled to 120 × 100 pixels.

Tongue images were processed as shown in Figure 1. The original image acquired by the tongue analyzer, which depicts the subject's entire face (Figure 1(a)), was first segmented to include only the rectangular area depicting the tongue (Figure 1(b)). Each image was then scaled to 120 × 100 pixels (Figure 1(c)) to enable efficient feature extraction while retaining color information.

Several feature types can be used in image analysis. Features based on statistical analysis of color represent global differences (expressed as means and standard deviations) among images and are the most intuitive feature type [15], but they cannot describe differences among areas in a single image. The use of pixel analysis to define image features has a high computational cost and does not provide high-level information about the images [23]. Moreover, the number of pixels is much greater than the number of images in most situations, and adjacent pixels are often closely correlated; these characteristics complicate statistical analysis. For this reason, we used Haar-like features [23], which fall between the pixel and global levels, in the present study. These features enable examination of color differences between areas, partially solving the problem of correlations among pixels. However, the number of such features is large, exceeding 160,000 in a 24 × 24-pixel image, and the computational cost of Haar-like feature extraction remains large [23].

**Figure 2:**
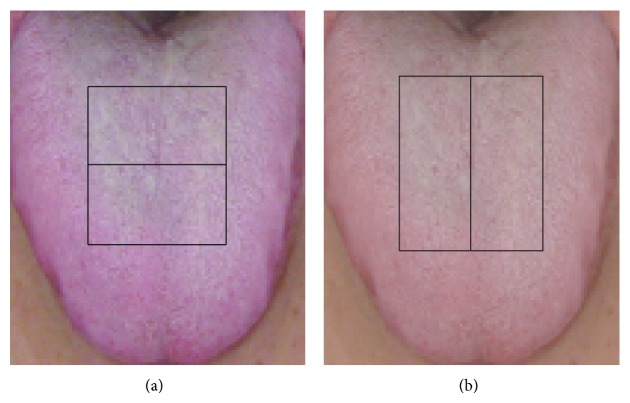
Upper-lower and left-right area arrangements. The sum of pixels in the lower (a) or right (b) rectangle is subtracted from the sum of pixels in the upper or left rectangle, respectively.

**Figure 3:**
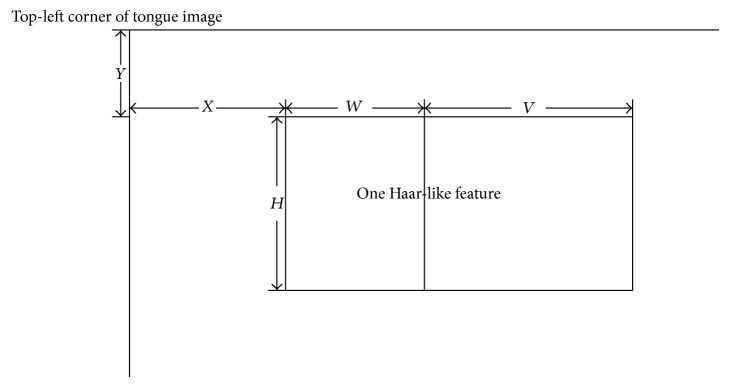
Haar-like feature parameters. *X* and *Y* describe the position of the top-left corner of the feature. *H* represents the height of a feature. *W* and *V* represent the widths of the left and right rectangles, respectively.

We first sought to reduce the number of Haar-like features in the tongue images, which exceeded our computing capabilities. Considering that observation of the tongue is based on color, we first selected features in the red, green, and blue color plains, ignoring plains in other color spaces (i.e., Lab). We then employed directional selection, which involved the delineation of two adjacent rectangles on each image.  Figure 2 shows two approaches to such selection: the sum of pixels in the lower or right rectangle may be subtracted from that in the upper or left rectangle, respectively. Given that the human body is characterized predominantly by bilateral symmetry, we subtracted the sum of pixels in the right from that in the left rectangle to select Haar-like features. Finally, we applied scale selection based on the five parameters of the left-right feature (Figure 3). Given that facial pixels near image boundaries were meaningless for this study, we determined the values of *Y* and *X* (which describe the position of the top left corner of a feature) as {11,12,…, 110} and {11,12,…, 90}, respectively. Given that overly narrow or short features provide inadequate information, we determined the values of *W* (width of left rectangle) and *H* (feature height) as {10,15,…, ⌊(100 − *X*)/2⌋} and {10,15,…, 100 − *Y*}, respectively. Considering the number of pixels contained in the two rectangles, we set *W* = *V* (width of right rectangle). Integral image scanning [20] was applied to reduce the computational cost of feature extraction. This technique requires a single image scan, and values of all features can be computed within several seconds. Because feature positions and scales are determined by *X*, *Y*, *W*, and *H*, features are identified by these values using the format “feature (*X*, *Y*, *W*, *H*)” in this text. Each color plain contained 195,840 features (total = 587,520 features in three plains).

### 2.3. Statistical Analysis

At this stage of processing, the number of selected Haar-like features far exceeds the number of subjects and correlation among features remains strong due to overlap, preventing direct application in classificatory models. Gorkani and Picard [27] found that human eyes distinguish images using high-level textural features. In this study, we thus assumed that the diagnosis of hyperuricemia would be based on instantaneous extraction of a feature from a tongue image in a single glance. We also assumed that all glances would be independent. We used Student's *t*-tests to examine the null hypothesis that *μ*_1_ = *μ*_2_, where *μ*_1_ and *μ*_2_ represent the mean values of one Haar-like feature in samples from the case and control groups, respectively. To speed up the calculation, we divided these data into four almost equal parts and ran tests on a personal computer (Lenovo M8000t; Quad Core, Q6600 CPU, 8 GB RAM). The statistical software used was R 2.15.2 [28].

Given recent suspicion of the discriminatory value of *P* < 0.05 [29] and the small deviation in mean values between features associated and not associated with hyperuricemia in comparison with their standard deviations, we investigated data dispersion using the following formula:

(1) $$\begin{array}{c} df=\frac{2|\mu_{1}-\mu_{2}|}{\sigma_{1}-\sigma_{2}}, \end{array}$$

where *μ*_1_ and *σ*_1_ are the mean value and standard deviation, respectively, of a feature associated with hyperuricemia; *μ*_2_ and *σ*_2_ are the corresponding values for a feature not associated with hyperuricemia.

We selected 50 features with smallest *P* and largest *df* values to serve as single classifiers in this study. We then tested the ability of these features to correctly classify case and control subjects. Receiver operating characteristic (ROC) analysis was performed and areas under the ROC curve (AUCs) were calculated. For features with the smallest *P* values, largest *df* values, and largest areas, we considered that a classifier would perform best when its Youden index was maximized. We also determined the sensitivity and specificity of these classifiers.

## 3. Results

### 3.1. Participant Characteristics

Blood samples from 1332/1437 eligible participants were analyzed to determine SUA concentration. The mean age of this population was 43.8 ± 11.9 (range, 19–87) years and the prevalence of hyperuricemia was 9.01% (120/1332; 13.0% in men and 3.15% in women). Application of the exclusion criteria left a sample of 83 subjects with and 211 without hyperuricemia. The final case-control-matched sample comprised 46 subjects (36 men and 10 women) per group. Mean age did not differ significantly between the case and control groups (46.2 ± 13.1 versus 45.2 ± 13.1 years; *P* = 0.763), but SUA concentration did (324 ± 81.8 versus 182 ± 32.7 *μ*mol/L; *P* = 4.18 × 10^−18^).

### 3.2. Haar-Like Features Useful for Hyperuricemia Diagnosis

**Figure 4:**
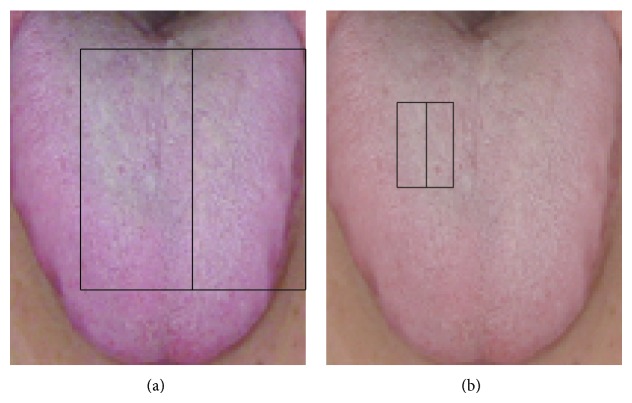
Three features on the red color plain examined in this study. (a) contains 6800 pixels. (b) contains 600 pixels.

**Figure 5:**
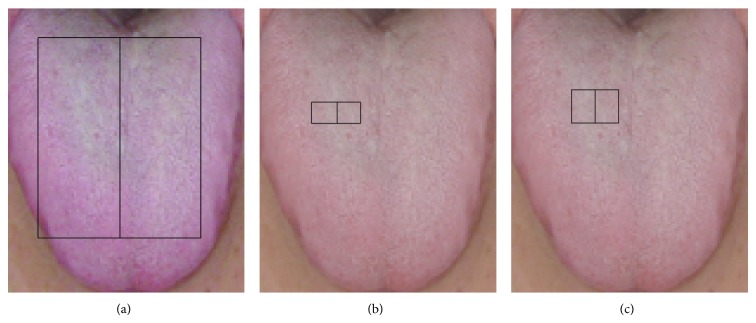
Three features on the green color plain examined in this study. Panel (a) contains 5950 pixels, (b) contains 300 pixels, and (c) contains 200 pixels.

**Figure 6:**
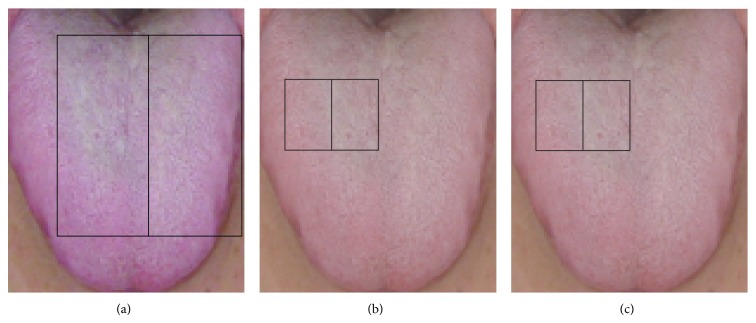
Three features on the blue color plain examined in this study. Panel (a) contains 6800 pixels, (b) contains 1200 pixels, and (c) contains 1200 pixels.

Initial screening of features using the criterion *P* < 0.05 yielded a sample of 239,035 features. Selection based on color plains reduced the number of features to several fractions of the original, yielding 97,124 features on the red plain, 26,228 features on the green plain, and 115,683 features on the blue plain that were useful for the diagnosis of hyperuricemia (all *P* < 0.05). The largest features on the red, green, and blue color plains, all of which were in the centers of tongue images, were feature (11,21,40,85) (*P* = 1.72 × 10^−2^; Figure 4(a)), feature (12,11,35,85) (*P* = 3.11 × 10^−2^; Figure 5(a)), and feature (11,21,40,85) (*P* = 1.19 × 10^−3^; Figure 6(a)), respectively (Table 1). The smallest *P* values were obtained for feature (30,23,10,30) (*P* = 2.09 × 10^−7^; Figure 4(b)), feature (33,24,10,15) (*P* = 1.16 × 10^−4^; Figure 5(b)), and feature (29,11,20,30) (*P* = 4.85 × 10^−11^; Figure 6(b)) on the red, green, and blue color plains, respectively (Table 1). Features with the largest *df* values in the red, green, and blue plains were feature (30,23,10,30) (*df* = 1.19; Figure 4(b)), feature (38,24,10,10) (*df* = 8.48 × 10^−1^; Figure 5(c)), and feature (30,11,20,30) (*df* = 1.58; Figure 6(c)), respectively (Table 1). Feature (30,23,10,30) in the red plain had both the smallest *P* value and largest *df* value.

### 3.3. Single Classifier Performance

**Figure 7:**
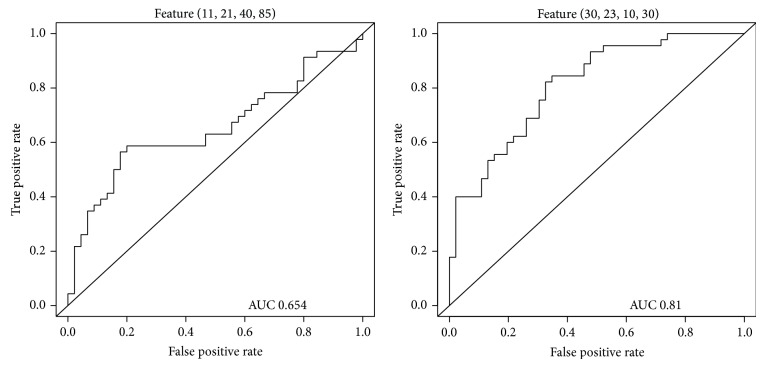
Receiver operating characteristic curves of features in the red plain.

**Figure 8:**
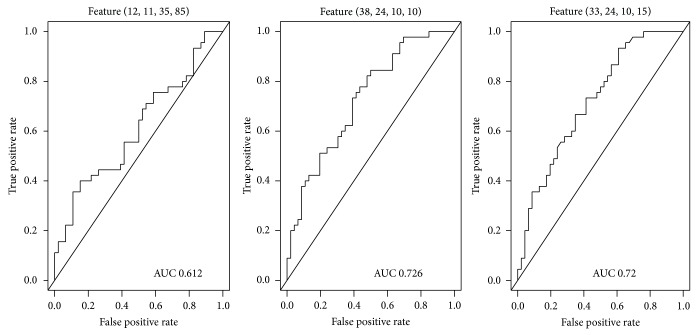
Receiver operating characteristic curves of features in the green plain.

**Figure 9:**
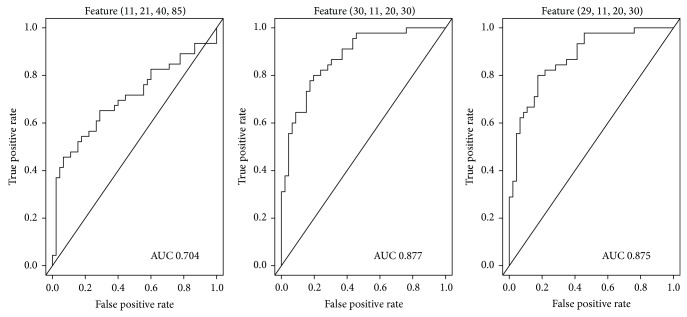
Receiver operating characteristic curves of features in the blue plain.

The ROC curves of the two features in the red plain are shown in Figure 7. The AUC of feature (11,21,40,85) was 0.654, preventing proper analysis of sensitivity and specificity. The AUC of feature (30,23,10,30) was 0.810; maximization of the Youden index yielded sensitivity and specificity values of 0.844 and 0.652, respectively. Features in the green and blue color plains showed similar characteristics. In the green plain, the AUC value of feature (12,11,35,85) was only 0.612, and those of feature (38,24,10,10) and feature (33,24,10,15) were 0.726 and 0.720, respectively (Figure 8). Maximization of the Youden index yielded sensitivity values of 0.500 and 0.844 and specificity values of 0.391 and 0.933 for the latter two features, respectively. These two features were also deemed to be inapplicable because of their low sensitivity values. In the blue plain, the AUC of feature (11,21,40,85) was 0.704 and those of feature (30,11,20,30) and feature (29,11,20,30) were 0.877 and 0.875, respectively (Figure 9). Sensitivity and specificity values for feature (29,11,20,30) were 0.800 and 0.804, respectively, when the Youden index was maximized. This feature achieved the best performance (sensitivity, 0.800; specificity, 0.826) when the maximum value of the Youden index was 0.626.

### 3.4. Cumulative Feature

**Figure 10:**
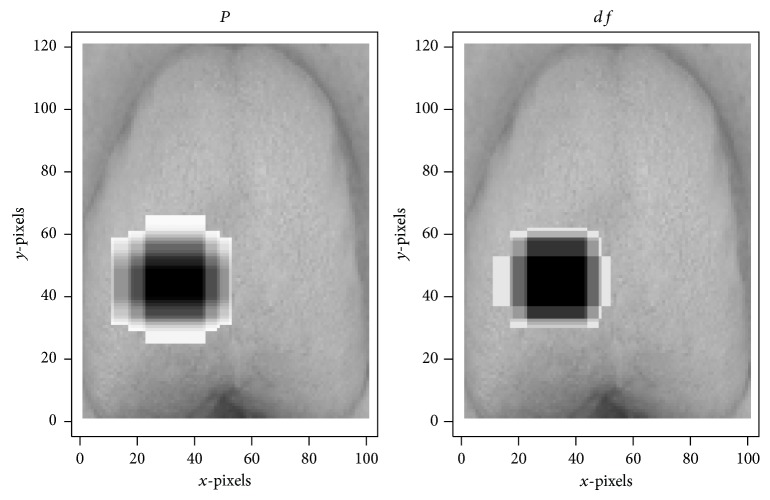
Cumulative image of 50 Haar-like features on the red plain. Note the cumulative feature's centralization around the tongue root.

**Figure 11:**
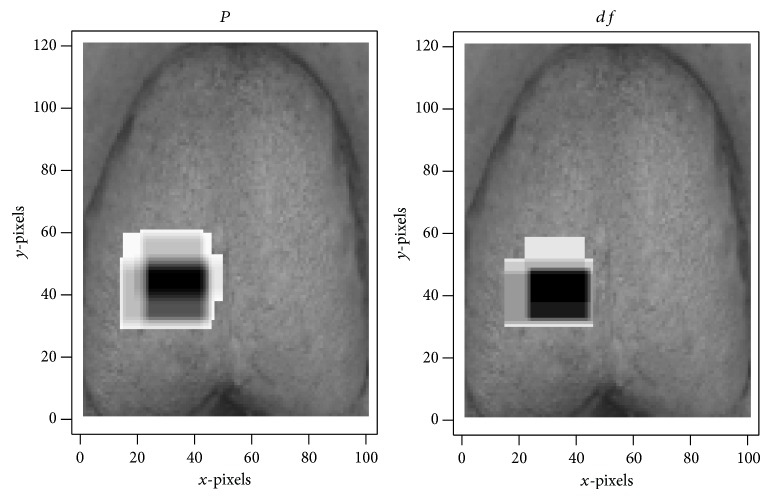
Cumulative image of 50 Haar-like features in the green plain.

**Figure 12:**
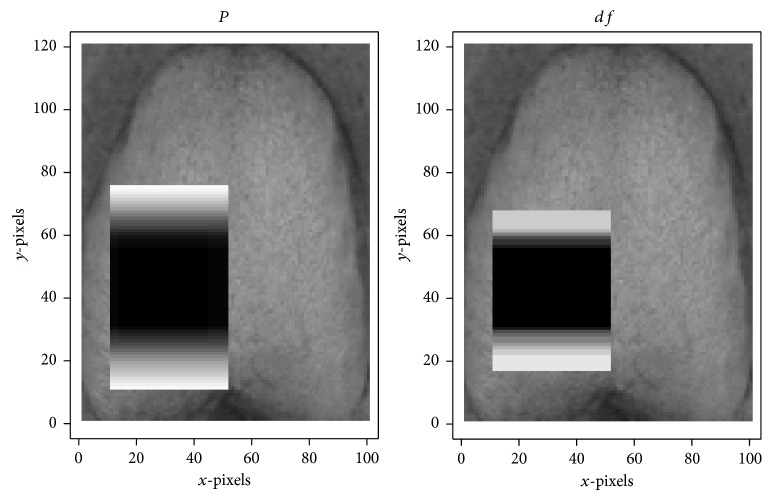
Cumulative image of 50 Haar-like features in the blue plain.

Cumulative features in the red, green, and blue color plains (each composed of 50 features) are shown in Figures 10, 11, and 12, respectively. All of these features were centralized around the tongue root, validating our hypothesis. The red cumulative feature has a circular distribution; the green cumulative feature is more concentrated than the red feature, and the blue cumulative feature shows vertical symmetry.

## 4. Discussion

Feature extraction is among the most important issues in image processing. These feature classes are based on perfect segmentation of a tongue image from the background, which is difficult for the human eye [14–16]. The extraction of Haar-like features does not require segmentation [20], greatly simplifying image preprocessing. In this study, we examined a rectangular area including the tongue, rather than attempting to perform more precise segmentation as in previous studies.

The use of Haar-like feature extraction from images is superior to extraction based solely on color because it allows the identification of local characteristics [19, 30, 31]. Other studies have focused on color differences of the entire tongue [17, 18] using global features, such as means and standard deviations of color value. These features prohibit detailed medical interpretation because they do not consider differences among parts of the tongue. In a previous study, the examination of tongue portions resulted in the identification of some features that were located outside of the tongue [16]. In our study, we scanned the entire tongue image and found that all meaningful features (those with the smallest *P* values and largest *df* values) were located within the tongue area. In contrast to those of previous studies, our results indicate that tongue image preprocessing does not require perfect tongue segmentation.

Tongue image preprocessing using Haar-like features is a new method that not only resolves the segmentation issue, but also provides a novel means of interpreting tongue images. A face detection study using Haar-like features provided the intuitive explanation that the most decisive features include the eyes and nose [23]. In our study, we found that the most decisive features for the diagnosis of hyperuricemia are centralized on the tongue root. A previous study described the results of tongue image analysis for the diagnosis of metastatic cancer [19], but applicable quantitative image analysis was not available at the time the study was performed, and the study also lacked a control group. Another study focused on elderly subjects [20]. In our study, we calculated quantitative feature values (*P* and *df*) to express differences between subjects with and without hyperuricemia using a case-control design and including subjects from all age groups.

The features identified in this study can be interpreted within the framework of TCM because they are based on pixels. All features that performed well in this study were centralized around the tongue root, the area considered to reflect kidney disease in TCM. This study provided direct evidence of the relationship between changes in the tongue root and kidney disease. The kidney filters blood and excretes metabolic waste products, including uric acid. In the human body, 70% of urate is disposed of* via* the kidneys [32]. Hyperuricemia is not only related to several diseases, but is also a risk factor for kidney injury [33–35]. The diagnosis of hyperuricemia thus provides early warning of kidney injury. However, the determination of serum urea nitrogen, creatinine, carbon dioxide, and uric acid concentrations requires time-consuming biochemical examination. An intuitive, inexpensive, and noninvasive method of hyperuricemia would thus be of benefit; TCM provides examination tools fulfilling these requirements. Our study provided direct evidence supporting the TCM method of diagnosing hyperuricemia based on tongue features.

However, this study has several limitations. First, the ROC analysis was performed using the same sample. In future studies, a test dataset will be collected to confirm the findings of this study. Second, given that the use of tongue images is a complementary and alternative diagnostic method, the method described in this study should be combined with other available variables associated with hyperuricemia, such as body mass index and alcohol intake. We plan to take this approach in a further study. Third, because the sample was carefully selected and patients with underlying diseases associated with hyperuricemia were excluded, the use of tongue images for the diagnosis of hyperuricemia should be restricted.

## 5. Conclusions

Haar-like features extracted from tongue images differed significantly between subjects with and without hyperuricemia. The locations of these features are consistent with interpretations of tongue appearance in TCM and Western medicine, indicating the existence of a relationship between tongue root color and hyperuricemia in our sample.

## Figures and Tables

**Table 1:** Parameters of eight Haar-like features potentially useful for hyperuricemia diagnosis.

Color plain	*P*	*df*	*X*	*Y*	*W*	*H*
Red	1.72*E* − 02	5.13*E* − 01	11	21	40	85
Red	2.09*E* − 07	1.19	30	23	10	30
Green	3.11*E* − 02	4.59*E* − 02	12	11	35	85
Green	1.16*E* − 04	8.52*E* − 01	33	24	10	15
Green	1.07*E* − 04	8.48*E* − 01	38	24	10	10
Blue	1.19*E* − 03	7.19*E* − 01	11	21	40	85
Blue	4.85*E* − 11	1.58	29	11	20	30
Blue	4.50*E* − 11	1.58	30	11	20	30

*X*  and *Y* describe the position of the top-left corner of the feature. *H* represents the height of a feature. *W* and *V* represent the widths of the left and right rectangles, respectively.

## References

[1] Choi H. K., Atkinson K., Karlson E. W., Willett W., Curhan G. (2004). Purine-rich foods, daily and protein intake, and the risk of gout in men. *The New England Journal of Medicine*.

[2] Kaneko K., Aoyagi Y., Fukuuchi T., Inazawa K., Yamaoka N. (2014). Total purine and purine base content of common foodstuffs for facilitating nutritional therapy for gout and hyperuricemia. *Biological and Pharmaceutical Bulletin*.

[3] Scott J. T. (1991). Drug-induced gout. *Baillière's Clinical Rheumatology*.

[4] Brandstäter A., Kiechl S., Kollerits B. (2008). Sex-specific association of the putative fructose transporter *SLC2A9* variants with uric acid levels is modified by BMI. *Diabetes Care*.

[5] Lai L.-H., Chou S.-Y., Wu F.-Y., Chen J. J.-H., Kuo H.-W. (2008). Renal dysfunction and hyperuricemia with low blood lead levels and ethnicity in community-based study. *Science of the Total Environment*.

[6] Liu Q., Li Z., Wang H. (2012). High prevalence and associated risk factors for impaired renal function and urinary abnormalities in a rural adult population from Southern China. *PLoS ONE*.

[7] Fang W.-G., Huang X.-M., Wang Y. (2006). A cross-sectional study of hyperuricemia in state-employees in Beijing: prevalence and risk factors. *National Medical Journal of China*.

[8] Kim Y. S., Guevara J. P., Kim K. M., Choi H. K., Heitjan D. F., Albert D. A. (2010). Hyperuricemia and coronary heart disease: a systematic review and meta-analysis. *Arthritis Care and Research*.

[9] Grayson P. C., Kim S. Y., LaValley M., Choi H. K. (2011). Hyperuricemia and incident hypertension: a systematic review and meta-analysis. *Arthritis Care and Research*.

[10] Li M., Hou W., Zhang X., Hu L., Tang Z. (2014). Hyperuricemia and risk of stroke: a systematic review and meta-analysis of prospective studies. *Atherosclerosis*.

[11] Carnethon M. R., Fortmann S. P., Palaniappan L., Duncan B. B., Schmidt M. I., Chambless L. E. (2003). Risk factors for progression to incident hyperinsulinemia: the atherosclerosis risk in communities study, 1987–1998. *American Journal of Epidemiology*.

[12] Liu B., Wang T., Zhao H. (2011). The prevalence of hyperuricemia in china: a meta-analysis. *BMC Public Health*.

[13] Krishnan E. (2014). Interaction of inflammation, hyperuricemia, and the prevalence of hypertension among adults free of metabolic syndrome: NHANES 2009–2010. *Journal of the American Heart Association*.

[14] Zhang B., Wang X., You J., Zhang D. (2013). Tongue color analysis for medical application. *Evidence-Based Complementary and Alternative Medicine*.

[15] Pang B., Zhang D., Wang K. (2005). Tongue image analysis for appendicitis diagnosis. *Information Sciences*.

[16] Kim J., Son J., Jang S. (2013). Availability of tongue diagnosis system for assessing tongue coating thickness in patients with functional dyspepsia. *Evidence-Based Complementary and Alternative Medicine*.

[17] Wang X., Zhang B., Yang Z., Wang H., Zhang D. (2013). Statistical analysis of tongue images for feature extraction and diagnostics. *IEEE Transactions on Image Processing*.

[18] Jung C. J., Nam J. H., Jeon Y. J., Kim K. H. (2014). Color distribution differences in the tongue in sleep disorder. *Evidence-Based Complementary and Alternative Medicine*.

[19] Friedlander A. H., Singer R. (1978). Renal adenocarcinoma of the kidney with metastasis to the tongue. *The Journal of the American Dental Association*.

[20] Amir K. A., Bobba R. K., Clarke B. (2006). Tongue discoloration in an elderly kidney transplant recipient: treatment-related adverse event?. *The American Journal Geriatric Pharmacotherapy*.

[21] Tang Q., Chen J., Li J. (2010). Analysis of the tongue characteristics of liver depression and kidney insufficiency pid patients. *China Modern Medicine*.

[22] Yang M., Li C.-D., Liang W.-N., Li H. (2011). Correlational study on pathological changes of liver qi depression and kidney deficiency, tongue coating exfoliative cells and sex hormones in perimenopausal syndrome. *China Journal of Traditional Chinese Medicine and Pharmacy*.

[23] Viola P., Jones M. J. (2004). Robust real-time face detection. *International Journal of Computer Vision*.

[24] Valbusa F., Bertolini L., Bonapace S. (2013). Relation of elevated serum uric acid levels to incidence of atrial fibrillation in patients with type 2 diabetes mellitus. *The American Journal of Cardiology*.

[25] Grimes D. A., Schulz K. F. (2002). An overview of clinical research: the lay of the land. *The Lancet*.

[26] Grimes D. A., Schulz K. F. (2005). Compared to what? Finding controls for case-control studies. *The Lancet*.

[27] Gorkani M. M., Picard R. W. Texture orientation for sorting photos ‘at a glance’.

[28] R Core Team (2012). *R: A Language and Environment for Statistical Computing*.

[29] Nuzzo R. (2014). Statistical errors. *Nature*.

[30] Kanawong R., Obafemi-Ajayi T., Yu J., Xu D., Li S., Duan Y. Zheng classification in traditional chinese medicine based on modified specular-free tongue images.

[31] Kanawong R., Obafemi-Ajayi T., Ma T., Xu D., Li S., Duan Y. (2012). Automated tongue feature extraction for ZHENG classification in traditional Chinese medicine. *Evidence-Based Complementary and Alternative Medicine*.

[32] Vitart V., Rudan I., Hayward C. (2008). SLC2A9 is a newly identified urate transporter influencing serum urate concentration, urate excretion and gout. *Nature Genetics*.

[33] Tomita M., Mizuno S., Yamanaka H. (2000). Does hyperuricemia affect mortality? A prospective cohort study of Japanese male workers. *Journal of epidemiology/Japan Epidemiological Association*.

[34] Fang J., Alderman M. H. (2000). Serum uric acid and cardiovascular mortality: the nhanes i epidemiologic follow-up study, 1971–1992. *Journal of the American Medical Association*.

[35] Toprak O., Cirit M., Esi E., Postaci N., Yesil M., Bayata S. (2006). Hyperuricemia as a risk factor for contrast-induced nephropathy in patients with chronic kidney disease. *Catheterization and Cardiovascular Interventions*.

